# Daratumumab, Bortezomib, and Dexamethasone for Treatment of Patients with Relapsed or Refractory Multiple Myeloma and Severe Renal Impairment: Results from the Phase 2 GMMG-DANTE Trial

**DOI:** 10.3390/cancers15184667

**Published:** 2023-09-21

**Authors:** Lisa B. Leypoldt, Maria Gavriatopoulou, Britta Besemer, Hans Salwender, Marc S. Raab, Axel Nogai, Cyrus Khandanpour, Volker Runde, Anna Jauch, Manola Zago, Peter Martus, Hartmut Goldschmidt, Carsten Bokemeyer, Meletios A. Dimopoulos, Katja C. Weisel

**Affiliations:** 1 Department of Hematology, Oncology and Bone Marrow Transplantation with Section of Pneumology, University Medical Center Hamburg-Eppendorf, 20246 Hamburg, Germany; l.leypoldt@uke.de (L.B.L.);; 2 Department of Clinical Therapeutics, School of Medicine, Alexandra General Hospital, National and Kapodistrian University of Athens, 11528 Athens, Greece; 3 Department of Hematology, Oncology, Immunology, Rheumatology, University Hospital of Tuebingen, 72076 Tuebingen, Germany; 4 Asklepios Tumorzentrum Hamburg, AK Altona and AK St. Georg, 22763 Hamburg, Germany; 5 Internal Medicine V, University Hospital Heidelberg, 69120 Heidelberg, Germany; 6 Medizinische Klinik m.S. Hämatologie, Onkologie und Tumorimmunologie, Charité—Universitätsmedizin Berlin, 12200 Berlin, Germany; 7 Department of Medicine A, Hematology, Oncology and Pneumology, University Hospital Münster, 48149 Münster, Germany; 8 Department of Hematology and Oncology, University Hospital Schleswig-Holstein and University of Lübeck, 23538 Lübeck, Germany; 9 Department of Hematology, Oncology and Palliative Care, Wilhelm-Anton-Hospital, 47574 Goch, Germany; 10 Institute of Human Genetics, University of Heidelberg, 69120 Heidelberg, Germany; 11 Center for Clinical Trials, University Hospital of Tuebingen, 72070 Tuebingen, Germany; 12 Department of Clinical Epidemiology and Applied Biostatistics, Eberhard Karls University of Tuebingen, 72076 Tuebingen, Germany; 13 Internal Medicine V and GMMG-Study Group, University Hospital Heidelberg, 69120 Heidelberg, Germany

**Keywords:** multiple myeloma, relapsed/refractory, renal impairment, hemodialysis, daratumumab, clinical trial

## Abstract

**Simple Summary:**

Impaired kidney function is a common complication of a certain blood cancer called multiple myeloma. Patients with severe kidney problems are usually left out of medical studies, so data on safety and efficacy of treatments are limited for these patients. The academic phase II GMMG-DANTE trial (NCT02977494) investigated a combination of drugs—daratumumab, bortezomib, and dexamethasone—in patients who had already tried other treatments before, and had severe kidney impairment. Even though the study had to end early, the results were promising. About 67% of the patients had their cancer respond to the treatment, and their kidney function improved. The treatment was overall well tolerated.

**Abstract:**

Renal function impairment (RI) is a common complication in multiple myeloma (MM). However, limited data exist on the safety and efficacy of anti-MM regimens in patients with severe RI, as these patients are frequently excluded from clinical trials. This investigator-initiated multicentric phase II GMMG-DANTE trial evaluated daratumumab, bortezomib, and dexamethasone (DVd) in relapsed or refractory (r/r) MM patients with severe RI. r/rMM patients with ≥1 prior treatment line and a GFR <30 mL/min/1.73 m^2^ or undergoing hemodialysis were eligible and received eight cycles of DVd followed by daratumumab maintenance. The trial closed prematurely after 22/36 planned patients. The primary endpoint was overall response rate (ORR). Median age of patients was 70 (range 55–89) years, with a median GFR of 20.1 mL/min/1.73 m^2^ (interquartile range, 9.4–27.3 mL/min/1.73 m^2^), and eight patients under hemodialysis. Median number of prior lines was two (range 1–10). The trial was successful, albeit with premature termination, as it met its primary endpoint, with an ORR of 67% (14/21). The rates of partial response, very good partial response, and complete response were 29%, 29%, and 10%, respectively (*n* = 6, 6, and 2). Fourteen patients (67%) achieved renal response. After median follow-up of 28 months, median progression-free survival was 10.4 months; median overall survival was not reached. Higher-grade toxicity was mainly hematologic, and non-hematologic toxicities ≥Grade 3 were mostly infections (24%). The prospective GMMG-DANTE trial investigating DVd exclusively in r/rMM patients with severe RI showed efficacy and safety to be comparable to data from patients without RI.

## 1. Introduction

Even in this era of novel drugs, renal function impairment (RI) remains one of the most common complications of multiple myeloma (MM) both in newly diagnosed and relapsed or refractory (r/r) disease with cast nephropathy being the most frequent mechanism of renal damage [1,2]. Estimates range from 20% up to 50% of MM patients presenting with acute renal failure at first diagnosis [1,3,4,5]. While the prognosis improved over the last decade with the introduction of novel therapeutics, it remains impaired for MM patients with RI compared to patients without. In a large retrospective analysis, Dimopoulos et al. showed that median overall survival (OS) for patients with severe RI (estimated glomerular filtration rate (eGFR) < 30 mL/min/1.73 m^2^) improved from about 18 months before the year 2000 to 32 months for the period after 2005. This improvement, however, remained disadvantageous compared to a median OS of 54 months for patients without RI [1]. One reason for the impaired survival is the still highly elevated risk of early mortality (defined as within 2 months of initiating treatment) of 12% for patients with severe RI versus 3% for mild or no RI which has not markedly improved over time [1]. Other reasons for impaired survival in this patient population include effective treatment combinations being less frequently used or used at reduced dose intensities, treatment being less well tolerated, and generally more impaired organ functions [1,6,7].

Proteasome inhibitors (PI), particularly the first-in-class PI bortezomib, are one of the recommended drug classes by the International Myeloma Working Group (IMWG), and are preferable for patients with RI due to their renoprotective effects, lack of dose adjustment in RI, and ability to induce rapid reduction in tumor load [1,8]. This is especially true in combination with dexamethasone, as high doses of corticosteroids upfront proved beneficial in rapid recovery of renal function [8,9].

The addition of daratumumab, the first anti-CD38-monoclonal antibody, to standard-of-care regimens both in newly diagnosed and r/rMM has led to deepened response rates and prolonged survival [10,11,12,13,14,15]. One of the key characteristics of anti-CD38 treatment is the rapid reduction in serum free light chains (sFLC), which is crucial to the recovery of renal function. The triplet combination of daratumumab, bortezomib, and dexamethasone (DVd) as pivotally introduced by the CASTOR trial led to the approval of the DVd triplet in routine care [12]. However, since patients with severe RI were excluded from the CASTOR trial, prospective clinical trial data on the efficacy and safety of DVd in MM patients with severe RI including dialysis was missing. Here, we present the primary analysis of the investigator-initiated, open-label, multi-center phase 2 prospective GMMG-DANTE trial (NCT02977494) evaluating daratumumab, bortezomib, and dexamethasone in r/rMM patients with severe RI including dialysis.

## 2. Materials and Methods

Study design and participants: This prospective phase II, open-label, multi-center GMMG-DANTE clinical trial was designed as a two-stage Minimax Simon design (see Appendix A for further details) and recruited patients in 6 German and 1 Greek centers from 2017 to 2020. Patients were enrolled by local site clinical research staff. Patients aged 18 years and older were eligible if they had r/rMM according to international consensus criteria with at least one prior treatment line and severe RI defined as eGFR <30 mL/min/1.73 m^2^ (calculated with the Modification of Diet in Renal Disease (MDRD) formula [16]) or undergoing hemodialysis. Patients needed a WHO performance status of ≤3, with 3 only being acceptable if due to MM. Further inclusion criteria were laboratory findings within defined ranges at inclusion (e.g., absolute neutrophil count ≥1000/µL, platelet count ≥70/nL or ≥50/nL if ≥50% myeloma cell bone marrow infiltration). Exclusion criteria included systemic amyloidosis and severe comorbidities as well as secondary malignancies (further criteria can be found in the Appendix A). Patients with significant peripheral neuropathy or neuropathic pain of Grade 2 or higher were excluded. Subjects must not have received prior anti-CD38-antibodies and must not have had any MM specific treatment within 14 days prior to treatment start except for emergency treatment with dexamethasone (up to 160 mg cumulative dose). All patients provided written informed consent. The trial was approved by the competent authorities and the Tuebingen University Ethics Committee. The trial is registered at clinicaltrials.gov (NCT02977494).

Procedures: All patients received up to eight 21-day cycles of DVd in the approved dosage and daratumumab maintenance thereafter. Bortezomib was administered subcutaneously at a dose of 1.3 mg/m^2^ on Days 1, 4, 8, and 11 of Cycles 1 through 8, dexamethasone was administered orally or intravenously at a dose of 20 mg on Days 1, 2, 4, 5, 8, 9, 11, and 12, for a total dose of 160 mg per cycle. Dexamethasone could be dose-reduced in subjects over the age of 75, underweight, or with prior intolerance. Daratumumab was administered at a dose of 16 mg/kg intravenously once weekly (Days 1, 8, and 15) during Cycles 1 to 3, once every 3 weeks (on Day 1) during Cycles 4 to 8, and once every 4 weeks thereafter until disease progression, withdrawal of consent, or unacceptable side effects. No dose modifications for daratumumab were allowed. Disease assessments were performed per IMWG Uniform Response Criteria [17]. Adverse events (AEs) were collected up to 30 days following last study treatment dose. Events were graded per National Cancer Institute Common Terminology Criteria for Adverse Events (version 4.03).

Outcomes: The primary endpoint was overall response rate (ORR). Key secondary endpoints were progression-free survival (PFS), overall survival (OS), safety and renal efficacy according to IMWG [8].

Statistical analysis: Statistical analyses were performed, and figures were created using the Statistical Package for Social Sciences statistical software (SPSS), version 26.0 (IBM Corp., Armonk, NY, USA), and Microsoft Excel for Mac, version 16.38 (Microsoft, Redmond, DC, USA). The primary endpoint ORR was determined as best response throughout the trial and was analyzed in the per protocol population using a Minimax Simon Design with 24 patients in the first and 12 patients in the second stage (Type 1 error: 0.05 one-sided, Type 2 error: 0.20, insufficient success rate: 20%, promising success rate: 39%). Prolongation to the second stage could be performed if at least 6/24 successes were observed. This procedure was chosen to additionally allow an early evaluation of toxicity in this vulnerable patient population. The study was successful if at least 12 successes were observed after 36 patients. The time-to-event-analyses (PFS, OS and time till progression (TTP)) were analyzed using the Kaplan–Meier estimator. All other measurements (e.g., lab values) were analyzed descriptively. Safety analyses included all patients with at least one study treatment dose. A safety listing including adverse events of all CTCAE-grade and Grade ≥3 is presented using MedDRA, multiple occurrences of same AE are counted once with highest CTCAE-grade.

Role of the funding source: This clinical trial is a non-commercial, investigator-initiated trial by the academic sponsor University Medical Center Hamburg-Eppendorf. It was co-financed and supported by provision of study drug by Janssen-Cilag (Neuss, Germany).

## 3. Results

### 3.1. Patients and Treatment

Twenty-two patients from six German and one Greek site were enrolled between January 2017 and January 2020. The trial was prematurely stopped in April 2020 due to inferior recruitment because of both the broad commercial use of DVd in r/rMM and the impacts of the emerging COVID-19 pandemic. However, at this stage, the success criterion of the applied design was already reached (see below).

All patients started DVd treatment, the per-protocol population included 21 patients since for one patient it was not possible to obtain a response due to early death.

Median age of included patients was 70 years with 12 male (57.1%) and 9 female (42.9%) patients. The median eGFR was documented at 20.1 mL/min (interquartile range (IQR) 9.4–27.3 mL/min) with 8 patients (38.1%) undergoing hemodialysis. Median number of prior lines was two (range 1–10).

Twenty patients (95.2%) received previous bortezomib-containing regimens of which six patients were bortezomib-refractory; nine patients (42.9%) received previous lenalidomide-containing regimens of which four showed lenalidomide-refractoriness; and six patients (28.6%) received prior stem cell transplant. ECOG performance status was distributed between ECOG 0 in seven patients (33.3%), ECOG 1 in ten (47.6%) and ECOG 2 in four (19%). While data on international staging system (ISS) were not available, cytogenetic data were available for ten patients (48%) of whom five showed any cytogenetic high-risk features with three showing ≥2 high-risk abnormalities with gain1q21 being the most frequent abnormality. In addition, 4/10 patients showed t (11;14). Most frequent disease type was light chain myeloma in eleven patients (52.4%), followed by IgG in seven (33.3%) and IgA myeloma in two patients (9.5%). Most patients showed additional comorbidities, the most common being arterial hypertension (*n* = 9, 42.9%). Patient characteristics are shown in Table 1. 

Thirteen patients (62%) completed all eight cycles of DVd and continued with daratumumab maintenance. Median duration of treatment was 28 weeks (range 3–110). Relative dose intensities were 89.5% for daratumumab, 83.2% for bortezomib, and 83.3% for dexamethasone.

### 3.2. Efficacy

**Figure 1:**
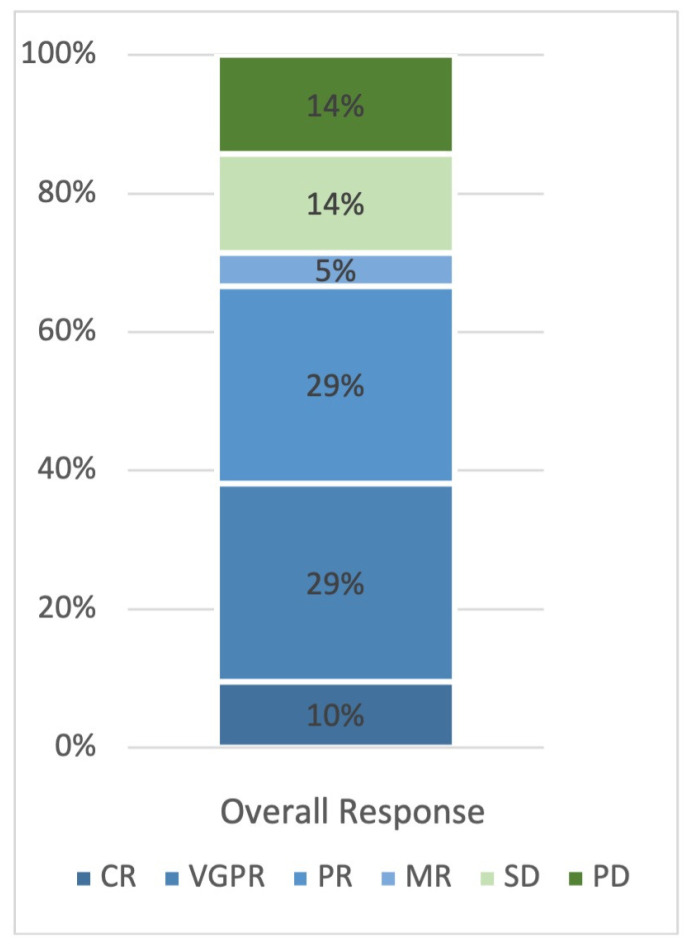
Overall response rate. CR, Complete response; VGPR, Very good partial response; PR, Partial response; MR, Minor response; SD, Stable disease; PD, Progressive disease.

At the time point of the analysis, all patients completed treatment. The trial met its primary endpoint with an ORR of 66.7% (14/21, where, according to the chosen Simon design, a minimum of 12 successes had to be observed for the trial to be successful). Six patients (29%) showed a partial response (PR), six patients (29%) showed a very good partial response (VGPR) and two patients (10%) showed a complete response (CR) (Figure 1). Median time to first response was 21 days, best response was reached after a median of 49 days. Of the eight patients under hemodialysis, three patients showed VGPR, two patients showed PR, and stable disease was seen in three patients, accounting for an ORR of this population of 62.5%. Overall, median treatment duration was 24 weeks (range 3–110). The reasons for discontinuation of the trial (“end of study”) were death in 10 patients, patient’s request (*n* = 2), withdrawal of consent (*n* = 1), lost to follow-up (*n* = 1) and, due to the premature closure, “sponsor decision to close the trial” for seven patients.

**Figure 2:**
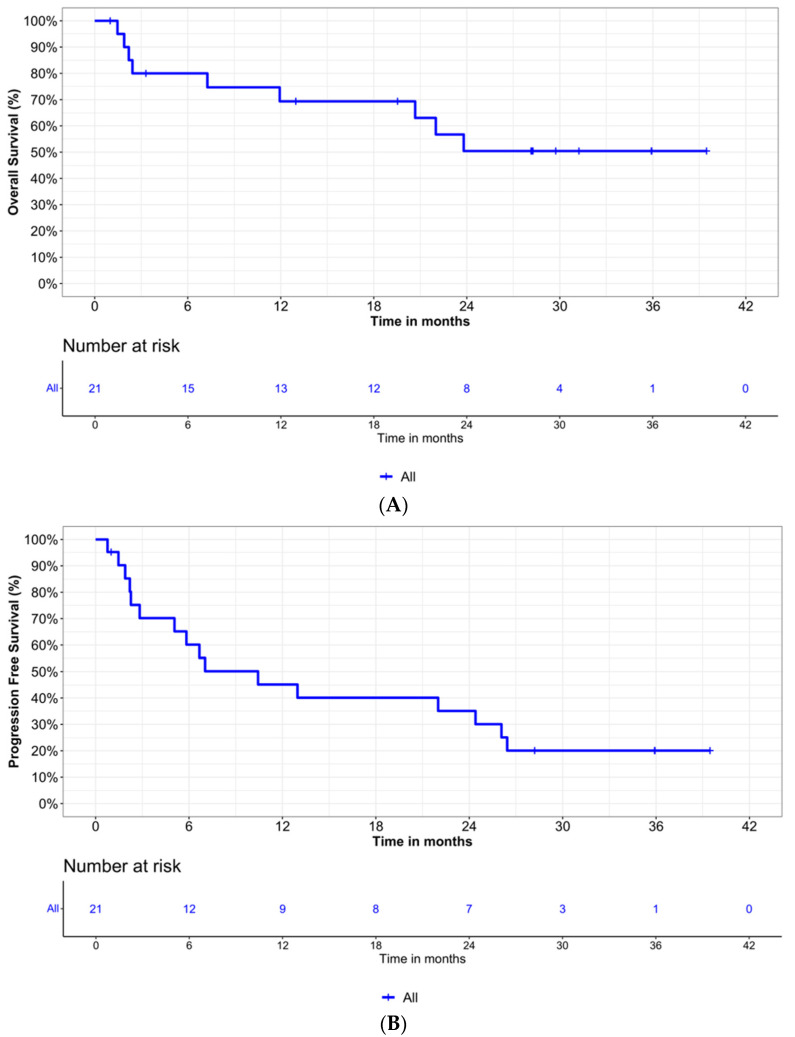
Kaplan–Meier plots for (**A**) overall survival and (**B**) progression-free survival.

After a median follow-up of 28 months, 16 (76.2%) PFS events and 9 (42.9%) OS events were documented with a median PFS of 10.4 months (Figure 2B). The Kaplan–Meier 12-month and 24-month PFS rates were 45% and 35%, respectively. The median OS was not reached; however, there was a plateau regarding OS after 24 months until 39 months (0.504) allowing for an estimated 2-year OS of 50% (Figure 2A). For patients under hemodialysis, a median PFS of 23 months was reported compared to 13 months in patients not under hemodialysis (Supplementary Figure S1A). Median OS was not reached for dialysis patients and was 21 months in patients not on dialysis (Supplementary Figure S1B).

In total, renal response according to IMWG [8] was achieved in 14 patients (66.7%) with 12 patients showing renal minor response (MRrenal, 57.1%), 1 patient showing PRrenal (4.8%) and 1 patient showing CRrenal (4.8%) responses. Overall, renal function showed improvement from an eGFR of 20.1 mL/min/1.73 m^2^ to 25.6 (±13.5) mL/min/1.73 m^2^ throughout the trial.

### 3.3. Safety

Treatment-emergent adverse events (TEAE) were mainly hematologic, with anemia in eight patients (38.1%), neutropenia in two (9.5%), and thrombocytopenia in eleven (52.4%). Grade 3/4 neutropenia was documented in one patient (4.8%), Grade 3/4 anemia and thrombocytopenia was documented in four patients each (19.0%) (Table 2).

Main non-hematologic adverse events (AEs) ≥Grade 3 were infections (*n* = 5, 23.8%) with mainly pneumonias (*n* = 3, including one RSV pneumonia). Polyneuropathy as an adverse event of special interest was described overall in 11 patients (52.4%), with 1 patient (4.8%) experiencing Grade 3/4 polyneuropathy (Table 2). Daratumumab-related infusion reactions were seen in four patients (19.0%), all of which were only mild (Grade 1–2) and occurred during the first doses (Table 2). There were no cases of tumor lysis syndrome and no permanent discontinuation of study drug due to an AE.

In 10 patients (47.6%), severe adverse events (SAE) were reported, the most frequent cause being again infectious complications (*n*= 5, 23.8%), mainly with pulmonal foci. A detailed list of all SAE is given in Table 3.

Throughout the trial and follow-up, two patients (9.5%) were diagnosed with secondary primary malignancies (one basal cell carcinoma, one melanoma in situ). Death on study occurred in 10 patients: five deaths were related to adverse events (four infections (RSV pneumonia; pneumogenic sepsis; sepsis without focus; diarrhea), one posterior reversible encephalopathy syndrome (PRES)), five deaths were not related (two due to progression of MM, two unknown and onw malignant arrhythmia, the latter in a patient with a history of Long-QT-syndrome, ICD implantation and dilated cardiomyopathy).

## 4. Discussion

Although renal function impairment is one of the most frequent complications in MM, only limited data are available for prospective evaluation of efficacy and safety of treatment combinations in this difficult-to-treat patient population. Clinical trials dedicated specifically to this indication are scarce, and patients are mostly excluded from many clinical trials not fulfilling renal inclusion criteria. The investigator-initiated GMMG-DANTE trial aimed to fill this gap by evaluating the efficacy and safety of the DVd triplet combination in r/rMM patients with severe RI and end-stage renal disease (GFR < 30 mL/min/1.73 m^2^ or undergoing dialysis). With a documented ORR of 66.7% and a PFS of 10.4 months, the trial showed the combination of daratumumab, bortezomib, and dexamethasone to be effective in MM patients with severe RI and under hemodialysis. In addition, stabilization to slight improvement of renal function was demonstrated, which is of high clinical importance.

So far, limited data exist on efficacy and safety data from other treatment regimens evaluated in a comparable patient population. A recent meta-analysis on randomized controlled trials conducted over a period of 15 years also highlighted the lack of clear reporting in the proportion of patients with RI enrolled in MM clinical trials [18]. The phase II MM-013 trial prospectively evaluated the combination of pomalidomide and dexamethasone in patients with renal impairment including hemodialysis [19]. Compared to our trial cohort, MM-013 trial patients were more heavily pretreated with a median number of four prior lines. In the MM-013 cohorts with a GFR < 30 mL/min/1.73 m^2^ and in the hemodialysis cohort (*n* = 34 and *n* = 14), ORR was 32.4% and 14.3% with a median PFS of 4.2 and 2.4 months, respectively. For monoclonal antibodies, data on patients undergoing dialysis from prospective trials are similarly scarce, although one of the key characteristics of these drugs is the achievement of a rapid decrease in the toxic sFLC with the advantage of no dose adjustment due to RI. The recently published DARE study (NCT03450057) was the first to prospectively report on daratumumab plus dexamethasone use in this population, with an ORR of 47.4% and a median PFS of 11.8 months [20], which further supports the results of our trial. A recent subgroup analysis from the ICARIA-MM trial investigating isatuximab, pomalidomide, and dexamethasone (Isa-Pd) in r/rMM, patients with RI (<60 mL/min/1.73 m^2^) showed a median PFS of 9.5 months; however, this trial only included patients with a GFR of ≥30 mL/min/1.73 m^2^ [21]. In fact, for the subgroup of patients with a GFR of 30–59 mL/min/1.73 m^2^, median PFS was shortened to 7.5 months, compared to 12.3 months for patients without RI [21], indicating the poor influence of RI on survival. Equally limited are data for the combination treatment of proteasome inhibitors and anti-CD38 antibodies in patients with severe RI. In the subgroup analysis of renally impaired patients included in the IKEMA trial investigating combination treatment of isatuximab, carfilzomib, and dexamethasone in patients with 1–3 prior lines, only three and four patients, respectively, showed a GFR >15 and <30 mL/min/1.73 m^2^ despite inclusion criteria allowing patients with a GFR >15 mL/min/1.73 m^2^ to be included [22]. To the best of our knowledge, the GMMG-DANTE trial is the first prospective clinical trial investigating an anti-CD38 monoclonal antibody triplet combination in r/rMM patients with severe RI including hemodialysis which underlines the importance of clinical trials specifically for, or not excluding, this patient population. With an ORR of 67% and a median PFS of 10.4 months, to our view, our data compare favorably to data published for this indication.

The safety profile of the triplet combination was acceptable. Given the knowledge about anti-CD38-antibody treatments and cytopenia as well as potential immune effects [12,14,23], close monitoring and adequate supportive treatments for treatment-emergent, particularly infectious adverse events are warranted. In this fragile patient population with a median age of 70 years and severely impaired renal function, prophylactic anti-infectives are key and substitution of immunoglobulins in case of a shortage thereof should be recommended to prevent infections. While the absolute numbers of TEAEs are within the expected range, the causes for deaths are indicative of a higher frailty of this patient population known for its elevated early mortality. Interestingly, patients under dialysis showed better overall survival than patients with severe RI not yet requiring dialysis, possibly indicating the latter population to be at even higher risk for complications. However, due to the small number of patients, this statement cannot be generalized without being verified on a larger scale. As expected, polyneuropathy as a common TEAE of bortezomib treatment was documented frequently, but could be limited to mostly lower grades.

While this trial is one of the few specifically aimed at the underrepresented population of MM patients with severe RI, it still has some limitations. Partly due to premature closure of the trial, which itself is an expression of the challenges recruiting this population, especially after approval and wide availability of the triplet regimen, the patient number was limited. The challenge to recruit patients with severe RI into clinical trials was recently also documented by Ramasamy et al. who reported 57 screen failures out of 88 screened patients for their OPTIMAL trial for newly diagnosed MM patients presenting with renal failure [24]. In OPTIMAL, most screen failure were due to frailty of the patients [24], again highlighting the special need of this specific population, and possibly contributing to the lack of attractiveness to include these patients into clinical trials. In addition, at the time of the conception of the GMMG-DANTE trial, assessments of minimal residual disease (MRD) were not yet widely available, so data on MRD negativity could not be collected. Being novel at the time of the conception of the trial, DVd remains a standard-of-care treatment and serves as comparator in ongoing phase III trials, highlighting its importance even in the era of bispecific T-cell engagers or CAR T-cell treatments.

## 5. Conclusions

Overall, the GMMG-DANTE trial showed that DVd is a safe and efficacious treatment regimen in r/rMM patients with severe renal function impairment including dialysis leading to potential improvement of kidney function.

## Figures and Tables

**Table 1:** Patient characteristics.

Characteristic		
**Age**	Median (range) (years)	70 (55–89)
	≥75 years–*n* (%)	7 (33.3)
**Sex**	Male–*n* (%)	12 (57.1)
	Female–*n* (%)	9 (42.9)
**Median time since initial therapy**–years (range)		4.01 (0.23–11.41)
**ECOG performance status score**	0–*n* (%)	7 (33.3)
	1–*n* (%)	10 (47.6)
	2–*n* (%)	4 (19.0)
	3–*n* (%)	0
**Body mass index**–median (range) (kg/m^2^)		25.9 (17.3–35.9)
**High risk cytogenetics ^a^**	≥1 aberration	5 (50.0)
	≥2 aberrations	3 (30.0)
	Gain1q21	5 (50.0)
	t (4;14)	2 (20.0)
	Del (17p13)	2 (20.0)
**Type of myeloma**	Light chain–*n* (%)	11 (52.4)
	IgG–*n* (%)	7 (33.3)
	IgA–*n* (%)	2 (9.5)
	Missing	1 (4.8)
**GFR**–median (range; IQR) (ml/min/1.73 m^2^) ^b^		20 (3.9–30; IQR 9.4–27.3)
**Undergoing hemodialysis**–*n* (%)		8 (38.1)
**Prior treatment lines**–median (range)		2 (1–10)
**Prior bortezomib exposure**–*n* (%)		20 (95.2)
**Prior lenalidomide exposure**–*n* (%)		9 (42.9)
**Prior stem cell transplantation**–*n* (%)		6 (28.6)
**Comorbidities** *n* (%) ^c^	**Arterial hypertension**	9 (42.9)
	**Diabetes mellitus**	4 (19.0)
	**Anemia**	4 (19.0)
	**Hyperuricemia/gout**	3 (14.3)
	**Bone pain**	3 (14.3)

^a^ cytogenetic data were available for 10 patients, ^b^ GFR, glomerular filtration rate, calculated by Modified Diet in Renal Disease (MDRD) formula [16], ^c^ occurring in >2 patients.

**Table 2:** List of adverse events (AE) (listed are all AE ^a^ that occurred in ≥ 5% of patients).

Adverse Event	All Grades–*n* (%)	Grade ≥ 3–*n* (%)
**Hematologic**		
Anemia	8 (38.1)	4 (19.0)
Leukopenia	3 (14.3)	1 (4.8)
Neutropenia	2 (9.5)	1 (4.8)
Lymphopenia	8 (38.1)	5 (23.8)
Thrombocytopenia	11 (52.4)	4 (19.0)
**Infectious**		
Upper respiratory tract infection/bronchial infection/common cold	9 (42.9)	0
Pneumonia	3 (14.3)	3 (14.3)
Infection, Not specified	4 (19.0)	0
Sepsis	2 (9.5)	2 (9.5)
**Neurologic**		
Peripheral polyneuropathy	11 (52.4)	1 (4.8)
Concentration disorder	2 (9.5)	0
**Cardiovascular**		
Arterial hypertension	4 (19.0)	1 (4.8)
Edema	8 (38.1)	0
Dyspnea	5 (23.8)	0
Hypotension	2 (9.5)	0
**General condition**		
Pain/back pain/bone pain	10 (47.6)	2 (9.5)
Fatigue	8 (38.1)	1 (4.8)
Insomnia	3 (14.3)	0
Reduced general condition	3 (14.3)	0
Dizziness	3 (14.3)	0
Weakness	2 (9.5)	0
Cough	2 (9.5)	0
**Gastrointestinal**		
Nausea	7 (33.3)	0
Diarrhea	2 (9.5)	0
Dysgeusia	2 (9.5)	0
Meteorism	2 (9.5)	0
Vomiting	2 (9.5)	0
**Other**		
Infusion reaction	4 (19.0)	0
Secondary primary malignancy	2 (9.5)	1 (4.8)
Renal function impairment	2 (9.5)	2 (9.5)

^a^ except for pure laboratory abnormalities without clinical significance.

**Table 3:** List of severe adverse events (SAE) during the trial.

Severe Adverse Event	All Grades–*n* (%)	Grade ≥ 3–*n* (%)
**Infections (in total)**	5 (23.8)	4 (19.0)
Pneumonia	2 (9.5)	2 (9.5)
Sepsis	2 (9.5)	2 (9.5)
Influenza	1 (4.8)	0
**Back Pain**	1 (4.8)	1 (4.8)
**Diarrhea**	1 (4.8)	0
**Disc prolapse**	1 (4.8)	1 (4.8)
**Dyspnea**	1 (4.8)	0
**Femoral fracture**	1 (4.8)	1 (4.8)
**Gastritis**	1 (4.8)	1 (4.8)
**Gastrointestinal bleeding**	1 (4.8)	1 (4.8)
**Hematuria**	1 (4.8)	00
**Hypercalcemia**	1 (4.8)	1 (4.8)
**Lewy body dementia**	1 (4.8)	1 (4.8)
**Malignant arrhythmia**	1 (4.8)	1 (4.8)
**Posterior reversible encephalopathy syndrome**	1 (4.8)	1 (4.8)
**Progressive disease**	1 (4.8)	0
**Renal function impairment**	1 (4.8)	0

## Data Availability

The original data for this paper are available upon reasonable request to the corresponding author, after publication.

## Supplementary Materials

The following supporting information can be downloaded at: https://www.mdpi.com/article/10.3390/cancers15184667/s1, Figure S1: Kaplan–Meyer curves for survival by dialysis dependency. (A) Progression-free and (B) overall survival of patients under hemodialysis and patients not on hemodialysis.

## Appendix A

Additional information on material and methods:

Study design: A two-stage Minimax Simon design (https://brb.nci.nih.gov/brb/samplesize/otsd.html (accessed on 7 August 2023)) was applied for this trial with 24 patients in the first stage and 12 patients in the second stage. The success probability P1 of a favorable drug was set to 0.39, the success probability P0 of an unfavorable drug was set to 0.20. In this design, with Type 1 error 0.05 (one-sided) and Type 2 error of 80%, a decision is possible regarding conducting a subsequent randomized phase 3 trial. In the first stage, six successes were necessary to prolong to the second stage; in total, 12 successes after Stage 2 were necessary to justify a subsequent phase 3 trial. In exceptional cases, stopping for success is possible even after the analysis of the first stage of the Simon design if the number of patients with a favorable result observed in the first stage is at least as large as the number of favorable results required in the combination of Stage 1 and Stage 2 (non-stochastic curtailing).

Participants: Additional inclusion criteria were laboratory findings within defined ranges at inclusion: total bilirubin ≤ 2.0 mg/dL and alanine and aspartate aminotransferase ≤2.5 times the upper limit of normal (ULN). Exclusion criteria also included severe cardiac (e.g., myocardial infarction within 6 months before date of study entry, unstable angina, cardiac insufficiency New York Heart Association (NYHA) Class III–IV or uncontrolled arrhythmia), pulmonary (e.g., chronic obstructive pulmonary disease (COPD) with a forced expiratory volume in one second (FEV1) <50% of predicted normal), psychiatric, neurologic, or infectious (e.g., active, uncontrolled infection) comorbidities. Patients had to strictly adhere to a pregnancy prevention plan.

Procedures: For patients undergoing hemodialysis, daratumumab was administered on a day without hemodialysis with the maximum distance to the next dialysis and bortezomib was administered on dialysis days after dialysis and on non-dialysis days according to institutional practice.

Statistical Analysis: Safety analyses were based on preferred term-level of MedDRA coding; for hematologic treatment-emergent adverse events (TEAE), the following terms were summarized: leukopenia and white blood cell count decreased, lymphopenia and lymphocyte count decreased, neutropenia and neutrophil count decreased, thrombocytopenia and platelet count decreased. All treatment-related and serious AEs were followed until stabilization or reversibility.
