# Supplementary material for: Daratumumab, Bortezomib, and Dexamethasone for Treatment of Patients with Relapsed or Refractory Multiple Myeloma and Severe Renal Impairment: Results from the Phase 2 GMMG-DANTE Trial
Source: Cancers (Basel). 2023 Sep 21;15(18):4667. doi: 10.3390/cancers15184667 (PMC10526417; doi:10.3390/cancers15184667)
Supplement: Supplementary file 1 [file cancers-15-04667-s001.zip › cancers-2578340-supplementary.pptx]

## Slide 1
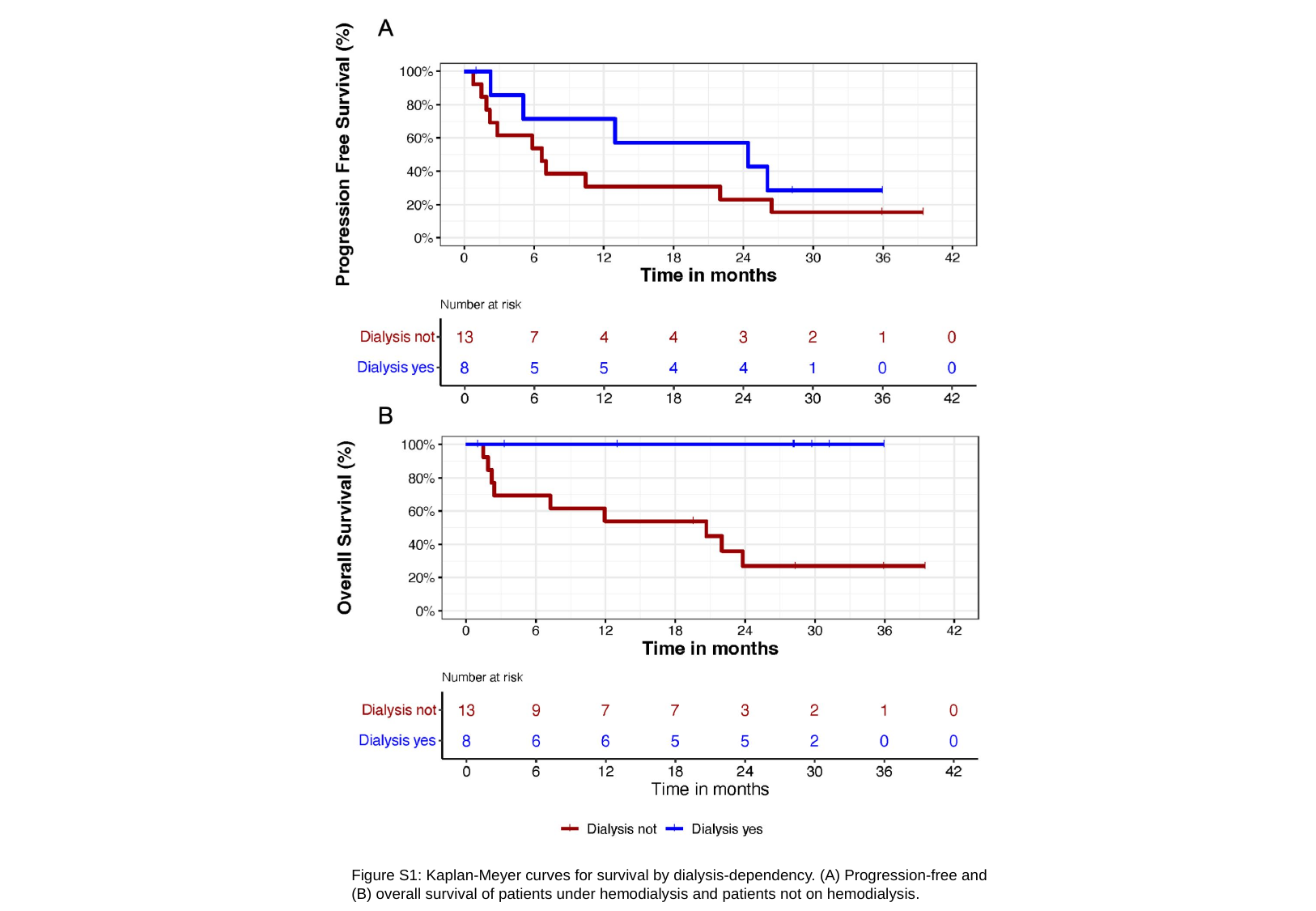

Figure S1: Kaplan-Meyer curves for survival by dialysis-dependency. (A) Progression-free and (B) overall survival of patients under hemodialysis and patients not on hemodialysis.
